# Copper-uptake is critical for the down regulation of synapsin and dynamin induced by neocuproine: modulation of synaptic activity in hippocampal neurons

**DOI:** 10.3389/fnagi.2014.00319

**Published:** 2014-12-03

**Authors:** Patricio A. Castro, Alejandra Ramirez, Fernando J. Sepúlveda, Christian Peters, Humberto Fierro, Javier Waldron, Sandra Luza, Jorge Fuentealba, Francisco J. Muñoz, Giancarlo V. De Ferrari, Ashley I. Bush, Luis G. Aguayo, Carlos M. Opazo

**Affiliations:** ^1^Department of Physiology and Membrane Biology, Shriners Hospital for Children Northern California, University of California at Davis School of MedicineCalifornia, USA; ^2^Laboratorio de Neurofisiología, Departamento de Fisiología, Facultad de Ciencias Biológicas, Universidad de ConcepciónConcepción, Chile; ^3^Oxidation Biology Laboratory, The Florey Institute of Neuroscience and Mental Health, The University of MelbourneParkville, Melbourne, Victoria, Australia; ^4^Laboratory of Molecular Physiology and Channelopathies, Departament de Ciències Experimentals i de la Salut, Universitat Pompeu FabraBarcelona, Spain; ^5^Center for Biomedical Research and FONDAP Center for Genome Regulation, Universidad Andrés BelloSantiago, Chile

**Keywords:** copper, zinc, neocuproine, synaptic activity, dynamin, synapsin, hyperexcitability, epileptiform-like activity

## Abstract

Extracellular and intracellular copper and zinc regulate synaptic activity and plasticity, which may impact brain functionality and human behavior. We have found that a metal coordinating molecule, Neocuproine, transiently increases free intracellular copper and zinc levels (i.e., min) in hippocampal neurons as monitored by Phen Green and FluoZin-3 fluorescence, respectively. The changes in free intracellular zinc induced by Neocuproine were abolished by the presence of a non-permeant copper chelator, Bathocuproine (BC), indicating that copper influx is needed for the action of Neocuproine on intracellular Zn levels. Moreover, Neocuproine decreased the mRNA levels of Synapsin and Dynamin, and did not affect the expression of Bassoon, tubulin or superoxide dismutase (SOD). Western blot analysis showed that protein levels of synapsin and dynamin were also down regulated in the presence of Neocuproine and that these changes were accompanied by a decrease in calcium transients and neuronal activity. Furthermore, Neocuproine decreased the number of active neurons, effect that was blocked by the presence of BC, indicating that copper influx is needed for the action of Neocuproine. We finally show that Neocuproine blocks the epileptiform-like activity induced by bicuculline in hippocampal neurons. Collectively, our data indicates that presynaptic protein configuration and function of primary hippocampal neurons is sensitive to transient changes in transition metal homeostasis. Therefore, small molecules able to coordinate transition metals and penetrate the blood-brain barrier might modify neurotransmission at the Central Nervous System (CNS). This might be useful to establish therapeutic approaches to control the neuronal hyperexcitabiltity observed in brain conditions that are associated to copper dyshomeotasis such as Alzheimer’s and Menkes diseases. Our work also opens a new avenue to find novel and effective antiepilepsy drugs based in metal coordinating molecules.

## Introduction

Zinc (Zn) and copper (Cu) ions have a key physiological importance in mammalian tissue (Mathie et al., 2006). Zn and Cu are abundant trace elements in the human brain (Takeda et al., 2001; Tarohda et al., 2004) and they participate in the regulation of brain physiology, being key structural components of several proteins and co-factors for enzymes that are critical for brain function, including enzymes involved in antioxidant defense and cellular respiration (Mathie et al., 2006). Increasing evidence suggests that Zn and Cu ions function as signaling molecules in the nervous system (Mathie et al., 2006). Moreover, these metals are released from the synaptic terminals of certain neurons, affecting postsynaptic receptors and regulating neuronal excitability (Hartter and Barnea, 1988; Kardos et al., 1989; Trombley and Shepherd, 1996; Vlachova et al., 1996; Weiser and Wienrich, 1996; Hopt et al., 2003; Peters et al., 2011; Opazo et al., 2014). Recently, some reports have described the effects of Cu at the synaptic level, where it modulates complex parameters such as LTP (Goldschmith et al., 2005; Gaier et al., 2013, 2014a,b) and receptor pharmacology (Peters et al., 2011; Marchetti et al., 2014). On the other hand, Zn has been considered to play a protective role, because zinc deficiency in the diet can cause malfunctions of several organs and physiological functions (Fukahori and Itoh, 1990). Zn-deficient animals display abnormalities in behavior, which is associated to the deregulation of Zn-binding ProSAP/Shank family members (Grabrucker et al., 2014). In addition, Zn can regulate different intracellular pathways that may explain the effect of Zn-deficiency in brain development (Mackenzie et al., 2011; Nuttall and Oteiza, 2012).

Metal chelators are usually used to evaluate the effect of metals on cell cultures (Calderaro et al., 1993; Göçmen et al., 2000). For example, Neocuproine, a membrane permeable Cu (I) chelator, has been frequently used as a protective agent against oxidative stress caused by Cu (Calderaro et al., 1993). Moreover, Neocuproine can inhibit the relaxation of electrically stimulated mouse corpus cavernosum (Göçmen et al., 2000) and the facilitation of bladder contraction induced by purinergic nerve stimulation (Göçmen et al., 2005).

Considering that hippocampal formation is enriched in transition metals (Tarohda et al., 2004), we evaluated the effect of Neocuproine on different synaptic parameters of primary hippocampal cultures. We observed that Neocuproine acutely increased intracellular Cu and Zn levels that lead to a concomitant decrease in the number of active neurons, an effect which was dependent of copper influx from the extracellular space. These changes correlated with a decrease in levels of synapsin and dynamin. Moreover, Neocuproine blocked the epileptiform-like activity induced by bicuculline in hippocampal neurons. Thus, the use of molecules that can modulate the free levels of Cu and Zn ions could have potential roles on the physiopathology of the central nervous system (CNS). For example, the information presented in this paper can be useful to establish therapeutic approaches to control hyperexcitability observed in brain conditions associated to copper dyshomeostasis such as Alzheimer and Menkes disease (Palop et al., 2003; Amatniek et al., 2006; Prasad et al., 2011; Schrag et al., 2011; James et al., 2012). This work also opens new venues to find novel and effective antiepilepsy drugs (Cully, 2014) based in metal coordinating molecules.

## Materials and methods

### Primary cultures of rat hippocampal neurons

Hippocampal neurons were obtained from 18-day old pregnant Sprague-Dawley rats and maintained for 10–14 days *in vitro* (DIV) as previously described (Peters et al., 2011). Animals were obtained from the animal house of Catholic University of Chile (Santiago, Chile). All animals were handled in strict accordance with NIH recommendations and approved by the appropriate committee of University of Concepción (Concepción, Chile).

### Electrophysiology

Experiments were performed in the “whole-cell” configuration using the internal and external solutions described below. Synaptic activity was recorded after a stable baseline was reached. Recording pipettes were pulled from borosilicate glass (WPI, Sarasota, FL) in a horizontal puller (Sutter Instruments, Novato, CA). Membrane currents were measured using an Axopatch-200B amplifier (Axon Instruments, Inc., Burlingame, CA) and an inverted microscope (Nikon, Eclipse, TE200-U, Japan). Data was collected, stored and analyzed using a data acquisition system card (Axon Instruments, Inc.) and the pClamp9 software (Axon Instruments, Inc.). For synaptic activity records, data was analyzed using the Minianalysis software, obtaining the frequency, amplitude and decay time of the records. All experiments were performed at room temperature (20–25°C) using a holding potential of −60 mV. Data are given as means ± S.E.M. and are obtained from at least 3 experiments.

### Solutions and drugs

The intracellular medium contained (in mM): 120 KCl, 2 MgCl_2_, 2 ATP-Na_2_, 10 BAPTA, 0.5 GTP, 10 HEPES (pH 7.4). The extracellular medium contained (in mM): 150 NaCl, 5.4 KCl, 2 CaCl_2_, 1 MgCl_2_, 10 glucose and 10 HEPES (pH 7.4).

### Immunofluorescence

Hippocampal cultures were grown in glass coverslips precoated with poly-lysine (1%). After the treatments, hippocampal neurons were washed with PBS (pH 7.4), and then fixed with paraformaldehyde (4%) at RT for 10 min. Then, the dish was washed again in PBS and neurons were permeabilized and blocked for 30 min with PBST (PBS + triton 0.1%) and BSA 10%. Then, cells were incubated with the following primary antibodies for 16 h: Anti-synapsin and anti-MAP 2 antibody (Chemicon) were used in a 1:100 dilution. Secondary antibodies conjugated with FITC or Cy3 were used for fluorescent staining (Jackson ImmunoResearch Laboratories, PA). All of them were used at 1:500 for 2 h. Finally, samples were mounted in fluorescent mounting medium (DAKO, CA, USA) and images were obtained under a Nikon Eclipse confocal microscope (Nikon, Japan) (60×, water immersion, NA 1.4).

### RT-PCR

Total RNA was extracted from primary rat hippocampal cell cultures using RNAsolv (Omega Biotek). Two μg of RNA were used to prepare cDNA using Stratascript kit (Stratagene). PCR was performed with specific forward and reverse primers (Genbank) (Table 1), using 20–40 cycles in a Biorad Thermal Cycler.

**Table 1:** **The list and sequence of primers used for RT-PCR analysis**.

Protein	Forward primer	Reverse primer
**Bassoon**	5′-CCCCCAACCACTGCTAACTA-3′	5′-CGAGCACAGAGGGGAAGTAG-3′
**Snap29**	5′-AGAGCTGTGGGCAGAGTGTT-3′	5′-ACTCCATGCACACAAACCAA-3′
**Synapsin**	5′-CACCGACTGGGCAAAATACT-3′	5′-TGTGCTGCTGAGCATCTCTT-3′
**Dynamin I**	5′-CAGGACAGGCCTCTTCACTC-3′	5′-CCGATGTTGTTGATGGTCAG-3′
**Dynamin II**	5′-ACCCCACACTTGCAGAAAAC-3′	5′-GGCTCTTTCAGCTTGACCAC-3′
**Tubulin**	5′-GCACTCTGATTGTGCCTTCA-3′	5′-ACTGGATGGTACGCTTGGTC-3′
**SOD1**	5′-GTCGTCTCCTTGCTTTTTGC-3′	5′-CACCTTTGCCCAAGTCATCT-3′

### Western blot

Hippocampal cells were incubated with Neocuproine (0–30 μM), culture media was removed and cells were washed with PBS and then homogenized in sample buffer containing: SDS (4%), Glycerol (12%), Phenol Red (0.0025%) and 10% β-mercaptoethanol in Tris-HCl (450 mM, pH 8.45). 20 μl of each homogenate (≈ 10 μg of total protein) was submitted to SDS electrophoresis in 10–20% Tricine gels. Protein bands were transferred onto nitrocellulose membranes, blocked with 5% milk and incubated with the following primary antibodies: monoclonal anti-synapsin, anti-dynamin (1:500, Chemicon) and α-tubulin (1:1000, Sigma) as internal control. Immunoreactive bands were detected with secondary antibodies conjugated with HRP (Santa Cruz Biotechnology) and visualized with ECL Plus Western Blotting Detection System (PerkinElmer).

### Zn^**2+**^ and Cu^**2+**^intracellular measurements

Hippocampal cells were loaded for 30 min at 37°C with FluoZin-3-AM (1 μM) or Phen Green (5 μM) (Invitrogen, USA) to measure Zn^2+^ or Cu^2+^, respectively. Then, cells were washed twice with external solution as described above and incubated under control or experimental conditions for a maximal time of 30 min at 37°C. Confocal images were acquired on middle cellular plane, with 20× and 60× magnification. Intracellular fluorescent signals were recorded with a CCD camera (SensiCam camera, PCO, Germany), with 200 ms of exposition and 2 s acquisition (Ex:Em; 490:510 nm).

### Analysis of intracellular calcium transients

Hippocampal cells were loaded with Fluo-3 AM (1 μM in pluronic acid/DMSO, Molecular Probes, Eugene, OR, USA) for 30 min at 37°C and then washed twice with external solution, as described above, and incubated under control or experimental conditions for a maximal time of 30 min at 37°C. Neurons were mounted in a perfusion chamber that was placed on the stage of an inverted fluorescent microscope (Eclipse TE, Nikon), equipped with a CCD camera (SensiCam camera, PCO, Germany) and xenon excitation lamp. Cells were subsequently excited for 200 ms each 2 s intervals and recorded during 5 min, using a Lambda 10-2 filter wheel (Sutter Instruments) and regions of interest (ROI) were simultaneously selected on several neuronal somata on each plate (Ex:Em; 480:510 nm) recording more than 10 cells in each experiment. Finally, calcium transients, as defined by their TTX sensitivity (Gu et al., 1994), were acquired and analyzed off line with Axon Instruments Workbench 2.2 software.

### Data analysis

Results are expressed as the mean ± SEM. Statistical significance was determined using One-way ANOVA. A level of * *p* < 0.05, ** *p* < 0.01 and *** *p* < 0.001 was considered statistically significant followed by the Bonferroni post test.

## Results

### Neocuproine induces a copper-mediated intracellular zinc increase

**Figure 1:**
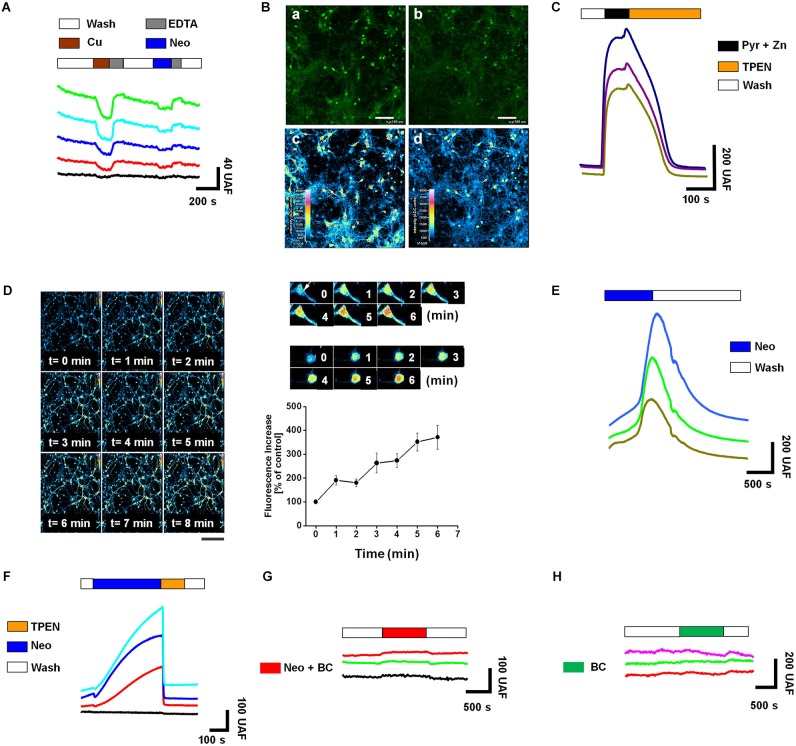
**Neocuproine increases intracellular Zn^2+^ [Zn^2+^]_i_ by promoting copper influx in hippocampal neurons**. **(A)** The traces show the intracellular changes of Phen Green fluorescence after extracellular acute application of CuCl_2_ (3 μM) (quenching), EDTA (10 μM) (recovery) or Neocuproine (Neo, 10 μM) (quenching). Traces in color correspond to the signal recorded in a single cell. The black trace corresponds to the typical background signal recorded outside of cells. **(B)** Confocal microphotographs of hippocampal cultures loaded with Phen Green, untreated (a and c) or treated with Neo (10 μM) for 10 min (b and d). Pseudocolor images shown in “**c**” and “**d**”, correspond to fluorescence photographs shown in “**a**” and “**b**”, respectively. Bar represents 100 μm. **(C)** The traces show the intracellular changes of FluoZin-3 fluorescence under control conditions or after the acute application of the agents indicated in the figure (Pyr/Zn = 30/10 μM; TPEN = 10 μM). Each trace corresponds to the signal recorded in a single cell.** (D)** Confocal microphotographs of hippocampal cultures loaded with FluoZin-3, before (time = 0) and after of a short application (up to 8 min) of Neo (10 μM). Scale bar, 250 μm. At the right of this panel is shown a confocal zoom of two neurons from the experiment described in “**(D)**”. The graph summarizes the intracellular Zn^2+^ changes recorded in the soma of single neurons treated as shown in “**(D)**”. Values are mean ± SEM (*N* = 3). **(E)** The traces show the rapid intracellular changes of FluoZin-3 fluorescence after the acute application of Neocuproine (Neo, 10 μM). **(F–H)** Each colored trace corresponds to the FluoZin-3 fluorescent signal recorded on the soma of single hippocampal neuron under the treatments indicated in the figures (Neocuproine, Neo = 10 μM; Bathocuproine, BC = 10 μM; TPEN = 10 μM). The black traces correspond to the typical background signal recorded outside of cells.

Zn and Cu may regulate the neurotransmission of CNS neurons by a presynaptic or postsynaptic mechanism (Goldschmith et al., 2005; Mathie et al., 2006; Leiva et al., 2009; Peters et al., 2011). Therefore, pharmacological intervention with compounds that change the balance of these metals at the neuronal level may result in changes in synaptic function. To test this idea we exposed primary rat hippocampal neurons (10–14 DIV) to Neocuproine (Neo), a high affinity copper chelator (Göçmen et al., 2000). First, we measured the changes of intracellular Cu using Phen Green, a fluorescent probe that decreases its fluorescent signal (quenching) when coordinates Cu (Chavez-Crooker et al., 2003). As it was expected, neurons loaded with Phen Green under control conditions displayed a stable basal fluorescent signal (Figure 1A). However, when they were exposed to CuCl_2_ (3 μM), the fluorescent signal was decreased, and a subsequent addition of EDTA (10 μM) to the bath recovered the signal to the basal levels (Figure 1A). Interestingly, when Neocuproine (10 μM) was added alone to the bath, a small decrease in the intracellular fluorescence was also observed, indicating that Neocuproine was inducing an increase of free intracellular copper (Figure 1A). This effect was observed in all neuronal cells exposed to Neocuproine and the changes were detected both in soma and neuronal projections (Figures 1Bb,d). These results suggest that changes in free intracellular Cu could be explained by a Cu influx from the extracellular space, occurring at different cell membrane domains, or by a Cu release from organelles or intracellular proteins, which are widely distributed at the intracellular space. In order to evaluate the effect of Neocuproine on the intracellular levels of other transition metal, we performed similar experiments using a specific fluorescent probe for Zn, FluoZin-3, which increases its fluorescence signal in the presence of Zn (Sensi et al., 2011). In fact, when Zn is co-applied with pyrithione (a Zn chelator) (Pyr/Z*n* = 30/10 μM) a rapid increase was observed (Figure 1C), which was abolished by a subsequent application of TPEN, a cell-permeant Zn chelator (Sensi et al., 2011; Figure 1C). Interestingly, when hippocampal cultures pre-loaded with Fluo-Zinc were exposed to Neocuproine (10 μM), the fluorescent signal rapidly increased (Figures 1D,E) in the soma and neuronal projections (Zoom on Figure 1D). We confirmed that this signal was associated with changes in free intracellular zinc, since the increase in the fluorescent signal induced by Neocuproine was abolished by a subsequent application of TPEN (Figure 1F). Therefore, Neocuproine increases simultaneously free intracellular Cu and Zn. Considering that Neocuproine has a higher affinity for Cu than Zn and also that the application of Neocuproine was at the bath solution, we hypothesized that Neocuproine facilitated the entry of copper from the extracellular space, which in turns displaced Zn from intracellular reservoirs. To test this hypothesis, we evaluated the effect of Bathocuproine (BC), a selective non cell-permeant Cu chelator (Mohindru et al., 1983), on the intracellular Zn changes induced by Neocuproine. In agreement with this hypothesis, we found that the presence of BC completely abolished the changes in Zn levels induced by Neocuproine (Figure 1G), indicating that Neocuproine facilitates the influx of copper, which then allows the rise of free intracellular Zn. We confirmed that BC itself was not changing the basal levels of intracellular Zn (Figure 1H). Altogether, these results indicate that it is possible to modify free intracellular Zn levels by using specific molecules that facilitate copper influxes from the extracellular space.

### Acute application of neocuproine does not affect synaptic activity

**Figure 2:**
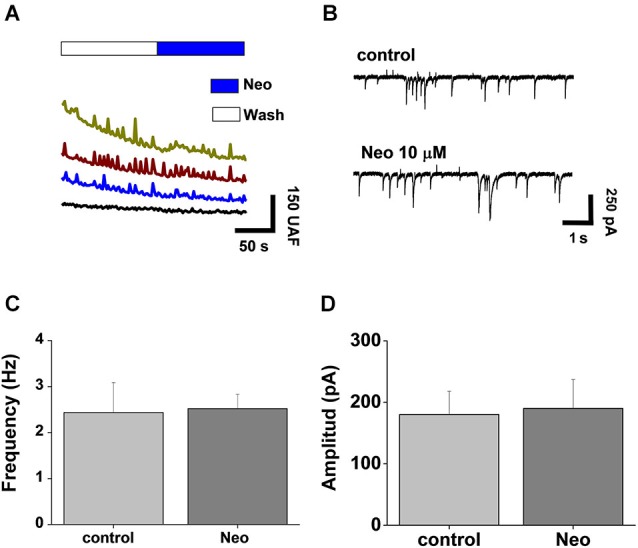
**Acute application of Neocuproine does not alter synaptic transmission**. **(A)** Calcium transients of hippocampal neurons in the absence or presence of Neocuproine (Neo, 10 μM). **(B)** Typical miniature synaptic currents obtained in the absence or presence of Neocuproine (Neo, 10 μM). The graphs summarize the frequency **(C)** and the amplitude **(D)** values of miniature synaptic currents recorded in three independent experiments performed under the conditions described in “**(B)**”. In both cases values are mean ± SEM (*N* = 3).

To evaluate if rapid intracellular changes in Cu and Zn induced by Neocuproine affects neurotransmission, we studied the synaptic activity of neurons under the same experimental conditions. Spontaneous calcium transients were recorded in the presence of the Fluo3-AM probe, allowing us to assess the changes on intracellular calcium as a reflection of synaptic and neuronal network activity (Peters et al., 2011). We observed no significant differences between control and acutely Neocuproine-treated neurons (Figure 2A). Moreover, when we evaluated the electrophysiological synaptic activity of these neurons by whole cell patch clamp recordings, the result correlated with calcium signals, since no differences were observed between control and Neocuproine-treated neurons (Figure 2B), in the frequency (control, 2.4 ± 0.6 Hz; Neocuproine, 2.5 ± 0.3 Hz) (Figure 2C) or amplitude (control, 280 ± 38 pA; Neocuproine, 190 ± 47 pA) (Figure 2D) of the currents. The patch clamp recordings also indicated that the control and Neocuproine-treated neurons presented an active neurotransmission.

### Sub-chronic application of neocuproine decreases the number of active neurons

**Figure 3:**
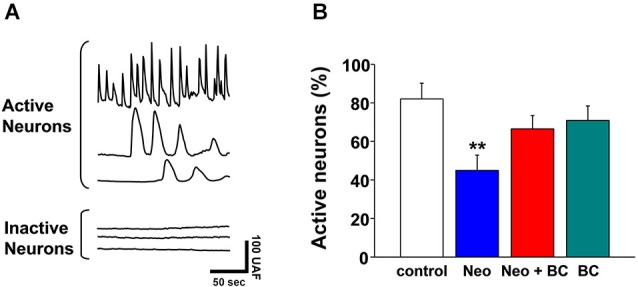
**Chronic application of Neocuproine decreases the number of active neurons**.** (A)** The top traces show typical calcium transients observed in active hippocampal neurons in culture. The bottom traces show the lack of calcium transients in inactive hippocampal neurons in culture. Each trace corresponds to the signal recorded in a single cell.** (B)** The graph summarizes the percentage of active hippocampal neurons under different treatments: untreated (control) or treated with Neocuproine (Neo, 10 μM) in the absence or presence of Bathocuproine (BC, 10 μM) for 12 h. Values are mean ± SEM (*N* = 3). **, *p* < 0.01.

To analyze if Neocuproine could induce changes in synaptic activity after sub-chronic incubations, hippocampal cultures were exposed to Neocuproine (10 μM) for 12 h and then the synaptic activity was evaluated measuring calcium transients. We found that the number of active neurons was decreased in the presence of Neocuproine, and that the effect was inhibited by the presence of BC in the cell media (Figures 3A,B), indicating that Neocuproine decreased the synaptic activity by a mechanism involving copper entry from the extracellular space.

### Sub-chronic application of neocuproine down-regulates synapsin and dynamin

**Figure 4:**
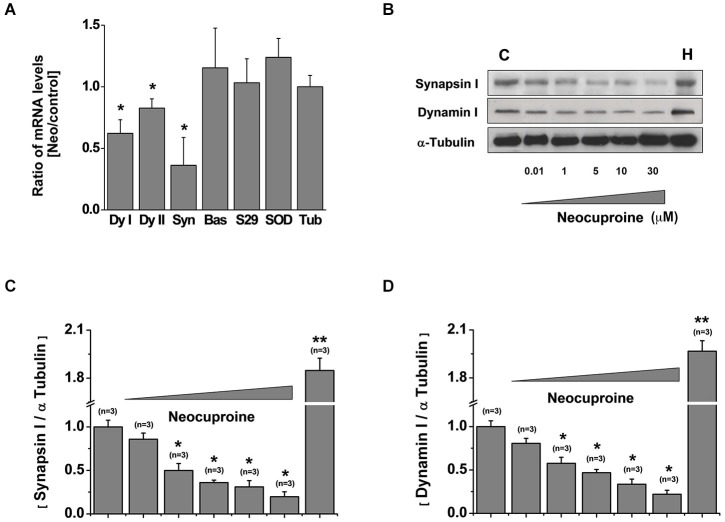
**Chronic application of Neocuproine down-regulates synapsin and dynamin. (A)** mRNA levels of dynamin I (dyI), dynamin II (dyII), synapsin, Bassoon (Bas), SNAP29, Superoxide Dismutase (SOD) and Tubulin (Tub) expressed by rat hippocampal cultures (11 DIV) treated in the absence or presence of Neocuproine (Neo, 10 μM) for 12 h. Values are mean ± SEM (*N* = 3). *, *p* < 0.05. **(B)** Protein levels of synapsin and dynamin I expressed by rat hippocampal cultures (11 DIV) treated in the absence or presence of Neo (up to 30 μM) for 12 h. Whole brain homogenate (H) of 15 days post-natal rat was used as positive control. **(C,D)** The graphs summarize the data obtained in experiments described in “**(B)**”. Values are mean ± SEM (*N* = 3). *, *p* < 0.05; **, *p* < 0.01.

**Figure 5:**
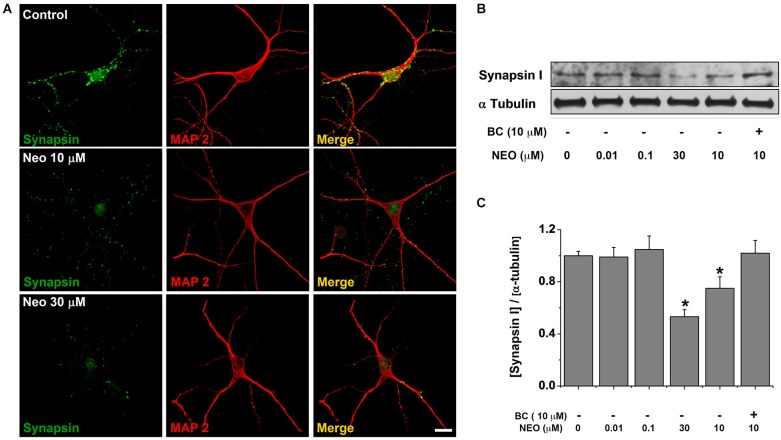
**Bathocuproine blocks the decrease of synapsin induced by Neocuproine treatments**. **(A)** Microphotographs of rat hippocampal neurons treated with Neocuproine (Neo, 10 μM and 30 μM) for 12 h. Synapsin immunoreactivity is shown in green, and MAP2 in red. Scale bar, 25 μm. **(B)** Protein levels of synapsin expressed by rat hippocampal cultures (11 DIV) treated in the absence or presence of Neo (up to 30 μM), and Neo (10 μM) + Bathocuproine (BC, 10 μM) for 12 h. **(C)** The graph summarize the data obtained in the experiment described in “**(B)**”. Values are mean ± SEM (*N* = 3). * *p* < 0.05.

We reasoned that the reduction on the number of active neurons after the treatment with Neocuproine should be accompanied with changes in the machinery responsible for neurotransmission, including scaffold and synaptic proteins. First, we analyzed the mRNA levels of dynamin I, dynamin II, synapsin, Bassoon, SNAP29, Superoxide Dismutase (SOD) and tubulin in rat hippocampal cultures treated in the absence or presence of 10 μM Neocuproine for 12 h. We found that the mRNA of dynamin (I and II) and synapsin, but not the mRNA of Bassoon and SNAP29 were decreased. We also did not observe changes in SOD or tubulin mRNA levels (Figure 4A). Then, hippocampal cultures were incubated with increasing concentrations of Neocuproine (up to 30 μM) for 12 h. Under these conditions protein levels for dynamin I and synapsin (Figures 4B–D) were decreased in the presence of Neocuproine in a concentration dependent manner. A similar pattern was also observed by immunofluorescence analysis (Figure 5A), indicating that Neocuproine decreased the signal of specific synaptic proteins such as synapsin. To evaluate if the change in synapsin levels induced by Neocuproine was also mediated by the uptake of copper from the extracellular media, we performed experiments in the presence of BC (Figures 5B,C). In agreement with this hypothesis, BC blocked the effect Neocuproine on synapsin levels, indicating that copper uptake was critical for the decrease in synapsin levels.

### Neocuproine blocks the epileptiform-like activity induced by bicuculline in hippocampal neurons

**Figure 6:**
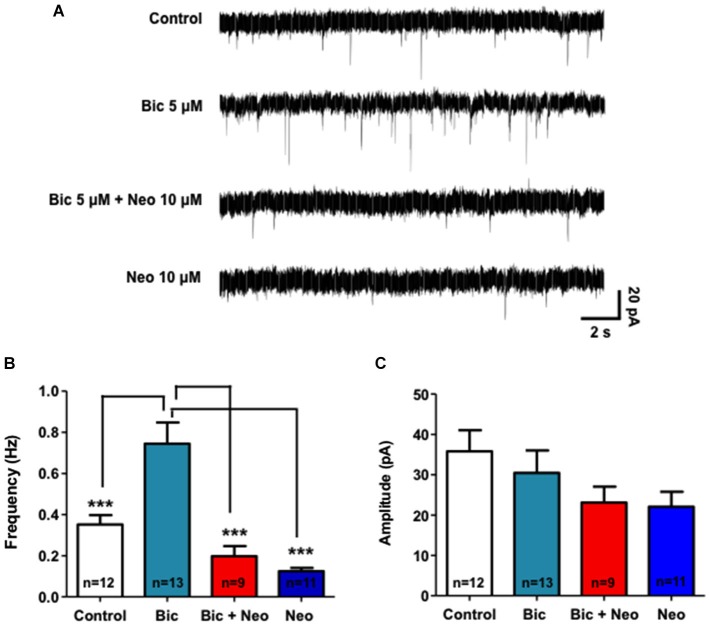
**Neocuproine blocks the epileptiform-like activity induced by bicuculline in hippocampal neurons. (A)** Representative traces of miniature postsynaptic currents illustrate the effect of control and chronic (24 h) bicuculline (5 μM) treatment in the absence or presence of Neocuproine (Neo, 10 μM) in hippocampal neurons. **(B,C)** The graphs summarize the effect of the different treatments on the frequency (Hz) and amplitude (pA) of the records obtained in three independent experiments performed under the conditions described in “**(A)**”. Values are mean ± SEM obtained from the indicated number of neurons. ***, *p* < 0.001.

Considering that Neocuproine modulates neurotransmission we evaluated whether Neocuproine down-regulates the epileptiform-like activity in hippocampal neurons. To evaluate the chronic effect of Neocuproine on the neuronal activity we used whole cell patch clamp technique (−60 mV) and measured miniature postsynaptic currents in primary hippocampal neurons treated with bicuculline for 24 h, which induce epileptiform-like activity in neurons (Carrasco et al., 2007; Sepúlveda et al., 2009). As shown in Figure 6, we found that the frequency of miniature postsynaptic currents was significantly lower after chronic administration (24 h) of Neocuproine (10 μM) compared to control (Control, 0.352 ± 0.045 Hz, *n* =12 vs. Neocuproine, 0.125 ± 0.016 Hz, *n* = 11; *p* < 0.01). However, no significant differences were observed in the amplitude of the miniature postsynaptic currents (control, 35.81 ± 5.22 pA, *n* = 12 vs. Neocuproine, 22.08 ± 3.72 pA, *n* = 11). These results correlate with the decrease in the number of active neurons (Figure 3) and the decrease on the levels of presynaptic proteins dynamin (I and II) and synapsin observed after sub-chronic treatments with Neocuproine (Figure 4). Interestingly, chronic treatments with Neocuproine blocked the epileptiform-like activity induced by bicuculline (5 μM; 24 h) (Figure 6; Control, 0.352 ± 0.045 Hz, *n* = 12; bicuculline, 0.744 ± 0.104; Neocuproine + bicuculline 0.197 ± 0.047; Neocuproine, 0.125 ± 0.016 Hz, *n* = 11; *p* < 0.01), suggesting that Neocuproine can abolish epileptogenic-like activity in CNS neurons.

## Discussion

Zn and Cu are abundant trace elements involved in the regulation of human brain physiology (Takeda et al., 2001; Tarohda et al., 2004), being key structural components of several proteins and co-factors for enzymes that are critical for brain function (Mathie et al., 2006). In fact, increasing evidence suggests that Zn and Cu ions function as signaling molecules in the nervous system (Mathie et al., 2006). Moreover, these metals are released from the synaptic terminals of certain CNS neurons, affecting postsynaptic receptors and regulating neuronal excitability (Hartter and Barnea, 1988; Kardos et al., 1989; Trombley and Shepherd, 1996; Vlachova et al., 1996; Weiser and Wienrich, 1996; Hopt et al., 2003; Peters et al., 2011; Sensi et al., 2011). Therefore, pharmacological strategies that interfere the homeostasis of these transition metals may modulate synaptic function. In this regard, this is the first time that Neocuproine, a high affinity copper chelator (Göçmen et al., 2000), is shown to decrease synaptic activity in hippocampal neurons (Figure 3), by a mechanism that involves the influx of Cu from the extracellular space that in turn induces the subsequent mobilization of intracellular Zn from proteins and/or organelles that were not identified in this study. According to our results, the presence of BC, a selective non cell-permeant Cu chelator (Mohindru et al., 1983), completely abolished the changes in the intracellular Zn levels induced by Neocuproine (Figure 1), indicating Neocuproine facilitated the influx of copper, which subsequently drove the rise of free intracellular Zn. In our working hypothesis, after Neocuproine-Cu complexes get across the plasma membrane Neocuproine donates Cu to Zn-proteins that interchange Zn and Cu in the intracellular space, thus releasing the Zn to the cytosol. We hypothesized that the rise of Zn induced by Neocuproine is occurring through a competitive mechanism. However, we could not discard that Neocuproine-Cu complexes or free Cu donated by Neocuproine-Cu complexes can inhibit, at the extra/intracellular space, Zn pumps or active Zn transporters that explain the rise in cytosolic Zn (Sensi et al., 2011). Moreover, all these changes in Cu and Zn homeostasis could promote the formation of reactive oxygen species (ROS), which may also participate in the regulation of synaptic proteins. We believe that our experimental conditions do not favor the formation of toxic levels of ROS, because the neuronal viability was not affected in the presence of Neocuproine. We acknowledge that different results can be expected in neuronal viability if exogenous Cu is added to the media, because it has been show that Neocuproine-Cu complexes are toxic to cultured astrocytes (Chen et al., 2008). However, we can not ruled out that non-toxic levels of ROS were produced in the presence of Neocuproine. Therefore, future studies are needed to better understand the mechanisms behind the effect of Neocuproine on living neurons, which may lead to establish protocols to change the neuronal activity. This information may be useful to prevent or to treat copper-related neurodegenerative diseases and neurological conditions, which remain untreatable to date (Bush, 2003). Interestingly, the effect of Neocuproine on free intracellular Cu and Zn was observed in cells maintained in cell media that was not supplemented with extra Cu or Zn and contained in total 1 μM of total Cu. Altogether, these results indicate that it is possible to modify the free intracellular Zn levels by using specific molecules that facilitate copper influxes from the extracellular space under basal conditions. These results also suggest that the alteration of the homeostasis of one transition metal can affect the homeostasis of another for a simple mechanism of competition.

We further found that the number of active neurons was decreased in primary cultures treated with Neocuproine, which was blocked by the presence of BC in the cell media (Figure 3), indicating that Neocuproine decreased the synaptic activity by a mechanism that involves the entry of copper to the cell from the extracellular space. Interestingly, the reduction on the number of active neurons after the treatment with Neocuproine was accompanied with changes in synapsin and dynamin I, both important proteins in the presynaptic machinery responsible for neurotransmission, through a direct or indirect mechanism that may lead to a reduction in neurotransmitter release. For example, Zn, Cu or Neocuproine-Cu complexes could directly interact with these proteins, decreasing their protein stabilities or/and their synthesis or/and increasing their degradation by the proteasome (Colledge et al., 2003). Because, we have recently found that Neocuproine does not change the ubiquitination pattern of neuronal cells (data not shown), a mechanism involving the ubiquitin-proteasome system can be partially ruled out, suggesting that the down-regulation effect of Neocuproine on dynamin and synapsin might be explained by changes in protein expression. All these possible mechanisms deserve further examination. In this regard, a protein involved in synaptic vesicle dynamic, SV2A, has been recently found to be an effective target for epilepsy (Gillard et al., 2011) supporting the presynapse as a target to control hyperexcitability.

In summary, in this work we have shown that Neocuproine, in time, concentration and copper dependent manner promotes changes on neurotransmission, suggesting that changes in transition metal might be required by neuronal cells to maintain adequate synaptic function (Peters et al., 2011). In fact, an increase in the brain concentration of copper, as well as a decrease in the levels of this metal, can lead to serious illness (Bush, 2003). Interestingly, Neocuproine blocks the epileptiform-like activity induced by bicuculline in hippocampal neurons, suggesting that Neocuproine might be a prototype drug to control the hyperexcitability observed in brain disorders such as Alzheimer and Menkes disease (Palop et al., 2003; Amatniek et al., 2006; Prasad et al., 2011), which are also characterized by brain copper dyshomeostasis (Schrag et al., 2011; James et al., 2012). Our data suggest that Neocuproine blocks the epileptiform-like activity by reducing the levels of synapsin and dynamin. However, we can not discard the participation of other presynaptic and postsynaptic targets that may be involved in the effect of Neocuproine on synaptic activity. Further *ex vivo* and *in vivo* studies are needed to confirm that Neocuproine is really effective to control hyperexcitability in humans. The origin of the brain overexcitation in these diseases is not well understood, but it might be related to changes in brain bioavailable copper. Interestingly, Tg2576 mice that overexpress APP and have lower levels of brain copper (Maynard et al., 2002) are more susceptible to chemical-induced seizures (Westmark et al., 2008), suggesting an association between brain copper homeostasis and brain overexcitation.

## Conflict of interest statement

The authors declare that the research was conducted in the absence of any commercial or financial relationships that could be construed as a potential conflict of interest.

## Abbreviations

BAPTA: 1,2-bis(o-aminophenoxy)ethane tetraacetate
BC: bathocuproine
CNS: central nervous system
DMSO: dimethyl sulfoxide
EDTA: Ethylenediaminetetraacetic acid
HEPES: 4-(2-hydroxyethyl)-1-piperazineethanesulfonic acid
ROS: Reactive Oxygen Species
SOD: Superoxide Dismutase
sPSCs: spontaneous postsynaptic currents
TPEN: N,N,N,N^′^-tetrakis-(2-Pyridylmethyl)ethylenediamine
TTX: tetrodotoxin.
